# Analysis of laboratory parameters before the occurrence of hepatic sinusoidal obstruction syndrome in children, adolescents, and young adults after hematopoietic stem cell transplantation

**DOI:** 10.1007/s00432-023-05561-w

**Published:** 2024-01-11

**Authors:** Lorena Johann, Bernd Gruhn

**Affiliations:** 1 Department of Pediatrics, Jena University Hospital, Am Klinikum 1, 07747 Jena, Germany; 2 Comprehensive Cancer Center Central Germany (CCCG), Jena, Germany

**Keywords:** Hematopoietic stem cell transplantation, Paediatric, Sinusoidal obstruction syndrome, Coagulation, Laboratory parameters

## Abstract

**Purpose:**

Hepatic sinusoidal obstruction syndrome (SOS) is a serious complication following hematopoietic stem cell transplantation (HSCT) in which early diagnosis improves patient outcome. The aim of our study was to detect laboratory parameters following HSCT that can predict the occurrence of SOS.

**Methods:**

This retrospective study included 182 children, adolescents, and young adults who underwent allogeneic or autologous HSCT for the first time (median age 7.2 years). The diagnosis of SOS was based on the pediatric criteria of European Society for Blood and Marrow Transplantation (EBMT). We investigated 15 laboratory parameters after HSCT before the onset of SOS.

**Results:**

The overall incidence of SOS was 14.8%. SOS developed in 24 of 126 allogeneic (19.1%) and in 3 of 56 autologous (5.4%) HSCT patients at a median time of 13 days after HSCT. We observed a low SOS mortality rate of 11.1% within 100 days after HSCT. International normalized ratio (INR) ≥ 1.3, activated partial thromboplastin time (aPTT) ≥ 40 s, reptilase time ≥ 18.3 s, factor VIII ≤ 80%, antithrombin III ≤ 75%, protein C ≤ 48%, D-dimer ≥ 315 µg/L, bilirubin ≥ 9 µmol/L, and ferritin ≥ 3100 µg/L showed significant associations with the onset of SOS in the univariate analyses. In the multivariate analysis, INR ≥ 1.3 [odds ratio (OR) = 8.104, *p* = 0.006], aPTT ≥ 40 s (OR = 10.174, *p* = 0.001), protein C ≤ 48% (OR = 5.215, *p* = 0.014), and ferritin ≥ 3100 µg/L (OR = 7.472, *p* = 0.004) could be confirmed as independent risk factors after HSCT before SOS. If three of the four significant cut-off values were present, the probability of developing SOS was more than 70%. The probability of SOS was 96%, if all four laboratory parameters were changed according to the cut-off values. The values of factor XIII, von Willebrand factor (vWF), von Willebrand factor activity (vWF activity), protein S, fibrinogen, and alanine aminotransferase (ALT) were not relevant for the occurrence of SOS.

**Conclusion:**

In summary, the laboratory parameters INR, aPTT, protein C, and ferritin were very useful to predict the occurrence of SOS. In addition, this is the first report on a significant association between SOS and high values of INR and aPTT after HSCT before SOS.

## Introduction

Hepatic sinusoidal obstruction syndrome (SOS), also known as veno-occlusive disease, is a serious complication after hematopoietic stem cell transplantation (HSCT), especially in children. The incidence of SOS following HSCT depends on diagnostic criteria, type of HSCT and patient age. Therefore the incidence varies in several publications. The median incidence of SOS is 14% and ranges from 0 to 60% in the published reports (Coppell et al. 2010; Xia et al. 2021). Following allogeneic HSCT the frequency of SOS is higher (12.9%) than after autologous HSCT (8.7%) (Coppell et al. 2010). A recent study by Coutsouvelis et al. (2023) reported an incidence of 11.5% in pediatric patients and 4.1% in adults. Further studies confirmed higher incidences in children (20%) than in adults (10%) (Barker et al. 2003; Cesaro et al. 2005; Corbacioglu et al. 2012, 2018).

Most patients with SOS suffer from hepatomegaly, ascites, and weight gain (Corbacioglu et al. 2018). There are several clinical diagnostic criteria used for SOS. Diagnostic standards are based on the Baltimore criteria (Jones et al. 1987) or the Seattle criteria (McDonald et al. 1984). In addition, new pediatric diagnostic criteria were published on behalf of the European Society for Blood and Marrow Transplantation (EBMT), stating that hyperbilirubinemia is a non-mandatory criterion and there is no time limit for the diagnosis of SOS (Corbacioglu et al. 2018). Furthermore, the pediatric EBMT criteria can be used for grading the severity of SOS, which consists of mild, moderate, severe, and very severe SOS (Corbacioglu et al. 2018).

Yoon et al. (2019) concluded an increased mortality of 36.7% within 100 days after HSCT in very severe SOS. Furthermore, Coppell et al. (2010) reported a mortality rate more than 80% in patients with the most severe SOS. Defibrotide is an effective therapy for SOS, which can improve survival (Mohty et al. 2022; Richardson et al. 2016). Mohty et al. (2022) reported better outcomes in patients with severe versus very severe SOS, suggesting early diagnosis and treatment is important before patients develop very severe SOS. In addition, defibrotide prophylaxis can reduce the incidence of SOS and is well tolerated by patients (Corbacioglu et al. 2012).

Different factors, such as conditioning therapy, endotoxins, cytokines, and certain drugs lead to endothelial imbalance. This results in a loss of sinusoidal endothelial cell fenestration and destruction of endothelial barrier. Erythrocytes penetrate into the space of Disse and damaged sinusoids induce vascular obstruction downstream (Carreras and Diaz-Ricart 2011; DeLeve et al. 2002). The reduced blood flow to the liver leads to liver damage (DeLeve et al. 2002). In addition, fibrinogen and factor VIII within the subendothelial zones can also lead to thrombosis of the vessels (Shulmann et al. 1987). Moreover, an exposure of tissue factors and initiation of the coagulation cascade, which is followed by reduced synthesis of coagulation factors, causes hemostatic imbalance (DeLeve et al. 2002; Nürnberger et al. 1997). Due to the pathogenesis, it is obvious that laboratory parameters, such as coagulation, fibrinolytic, and liver parameters are changed. An increase of D-dimer, bilirubin, and alanine aminotransferase (ALT) as well as a decrease of antithrombin III and protein C were observed in patients with SOS (Jevtić et al. 2011; Lee et al. 1998; Sartori et al. 2012).

The purpose of our retrospective study was to investigate laboratory parameters that can predict the development of SOS. Therefore, we analyzed coagulation, fibrinolytic, and liver parameters following HSCT before SOS.

## Methods

### Patients

Our study population consisted of 182 children, adolescents, and young adults who underwent HSCT at the Department of Pediatrics, Jena University Hospital, Germany between 2007 and 2022. The patients received allogeneic or autologous HSCT for the first time. We excluded patients with defibrotide prophylaxis and more than one HSCT, respectively. The conditioning therapy was always myeloablative and dependent on the type of HSCT and underlying disease. In addition, the transplanted patients underwent an infection prophylaxis and were isolated in rooms with special laminar airflow filtration systems.

### SOS

The pediatric EBMT criteria were used to diagnose SOS (Corbacioglu et al. 2018).

Accordingly, there is no time limit of onset and SOS is diagnosed if two or more of the following criteria are present: transfusion-refractory thrombocytopenia, unexplained weight gain > 5%, or weight gain on three consecutive days despite diuretics, ascites, hepatomegaly, bilirubin ≥ 34 µmol/L within 72 h, or an increase in bilirubin on three consecutive days. The classification of the severity of SOS was also based on the pediatric severity EBMT criteria (Corbacioglu et al. 2018). The patients with SOS received 25 mg/kg/day defibrotide in four single doses for 30 days.

### Risk factors

In our study, we considered the following 15 laboratory parameters: international normalized ratio (INR), activated partial thromboplastin time (aPTT), reptilase time, factor VIII, factor XIII, von Willebrand factor (vWF), von Willebrand factor activity (vWF activity), fibrinogen, antithrombin III, protein C, protein S, D-dimer, ALT, bilirubin, and ferritin. Laboratory parameters were measured weekly following HSCT. In the SOS patient group, the last laboratory values within one week before the onset of SOS were analyzed. We investigated the parameters for four weeks after transplantation in the non-SOS patient group and calculated the median.

### Statistical analysis

A retrospective analysis of potential risk factors was performed. We always considered results with *p* values less than 0.05 as statistical significant. Differences in the laboratory values between the SOS and the non-SOS patient group were analyzed using the Mann–Whitney *U* test. We performed univariate and multivariate analyses to find significant associations between the values of laboratory parameters and the occurrence of SOS. The results were presented with corresponding *p* value (*p*), odds ratio (OR), and 95% confidence interval (CI). Cut-off values were selected for the continuous parameters based on existing reference values and receiver operating characteristic (ROC) curves analyses. We divided the values according to the corresponding cut-off values and performed the univariate analyses by Fisher's exact test for nominal parameters. The significant variables were entered as independent variables into multivariate analysis, which carried out by binary logistic regression and backward elimination. Using the significant independent variables, we calculated the probability of developing SOS. All calculations were performed using the software IBM SPSS Statistics 27.

## Results

### Patient characteristics

This retrospective analysis included 182 patients (109 males and 73 females) with a median age of 7.2 years (ranged from 2 months to 26 years). Our study population consisted of 126 patients who underwent allogeneic HSCT (69.2%) and 56 patients who received autologous HSCT (30.8%). The most frequent underlying diseases were solid tumors (34.1%), genetic diseases (19.2%), acute lymphoblastic leukemia (18.1%), and acute myeloid leukemia (13.7%). Peripheral blood (50.5%) or bone marrow (49.5%) was used as stem cell source. Table 1 summarizes the characteristics of the patients.

**Table 1:** Characteristics of patients

Characteristics	No. (%)
Patients	182 (100)
Sex	
Male	109 (59.9)
Female	73 (40.1)
Median age, years (range)	7.2 (0.2–26.2)
Type of HSCT	
Allogeneic HSCT	126 (69.2)
Autologous HSCT	56 (30.8)
Stem cell source	
Peripheral blood	92 (50.5)
Bone marrow	90 (49.5)
Patient’s diagnoses	
Solid tumor	62 (34.1)
Genetic diseases	35 (19.2)
Acute lymphoblastic leukemia	33 (18.1)
Acute myeloid leukemia	25 (13.7)
Myelodysplastic syndrome	16 (8.8)
Lymphoma	9 (5.0)
Chronic myeloid leukemia	2 (1.1)
Conditioning regimen (based on)	
Chemotherapy	157 (86.3)
Total body irradiation	25 (13.7)
GVHD prophylaxis—allogeneic HSCT patients	
Cyclosporine A/methotrexate	55 (43.6)
Cyclosporine A	35 (27.8)
Others	36 (28.6)
SOS diagnosis	
Yes	27 (14.8)
No	155 (85.2)
SOS severity	
Mild	2 (7.4)
Moderate	7 (25.9)
Severe	8 (29.6)
Very severe	10 (37.1)

*HSCT* hematopoietic stem cell transplantation, *GVHD* graft-versus-host disease, *No.* number, *SOS* sinusoidal obstruction syndrome

A total of 27 patients (14.8%) developed SOS. In the allogeneic patient group, SOS was diagnosed in 24 of 126 patients, which corresponds to a frequency of 19.1%. The SOS frequency was lower in the autologous patient group, 3 of 56 patients developed SOS (5.4%). The time period between HSCT and the onset of SOS ranged from 1 to 28 days (median 13 days). Two patients (7.4%) showed a mild SOS and 7 patients (25.9%) a moderate SOS. Furthermore, a severe SOS occurred in 8 patients (29.6%). The remaining 10 patients (37.1%) developed a very severe SOS. Only 3 out of 27 patients, who suffered from very severe SOS, died within 100 days after HSCT (11.1%). Six out of 155 patients without SOS died in this time period (3.9%).

### Analysis of risk factors

Table 2 shows the medians and interquartile ranges of the investigated laboratory parameters as well as the results of the Mann–Whitney *U* test. The coagulation parameters INR, aPTT, and reptilase time demonstrated significantly higher values in the SOS group than in the non-SOS group. In Mann–Whitney *U* test, we detected that antithrombin III, protein C, and factor VIII were significantly lower in the SOS group than in the non-SOS group. Patients with SOS also had significantly higher levels of D-dimer, bilirubin, and ferritin. We observed no significant differences of factor XIII, vWF, vWF activity, fibrinogen, protein S, and ALT between the SOS group and the non-SOS group by using Mann–Whitney *U* test.

**Table 2:** Laboratory parameters with median, interquartile range, and *p* value of the Mann–Whitney *U* test

Laboratory parameters	SOS	No SOS	Mann–Whitney *U* test: *p*
INR			
Median	1.20	1.15	**0.006**
IQR	0.22	0.15	
aPTT in sec			
Median	42.00	34.50	** < 0.001**
IQR	6.00	5.50	
Reptilase time in sec			
Median	19.40	18.00	**0.012**
IQR	5.15	2.55	
Factor VIII in %			
Median	71.10	110.85	** < 0.001**
IQR	65.43	33.70	
Factor XIII in %			
Median	76.00	76.00	0.638
IQR	35.00	34.00	
vWF in %			
Median	156.10	180.15	0.367
IQR	91.25	61.41	
vWF activity in %			
Median	127.50	175.48	0.079
IQR	115.15	98.33	
Fibrinogen in g/L			
Median	3.70	3.45	0.946
IQR	1.60	1.25	
Antithrombin III in %			
Median	75.00	87.00	**0.001**
IQR	25.00	21.00	
Protein C in %			
Median	43.50	64.00	** < 0.001**
IQR	26.90	25.50	
Protein S in %			
Median	44.00	52.00	0.071
IQR	35.00	21.00	
D-dimer in µg/L			
Median	506.00	312.00	**0.006**
IQR	817.00	328.00	
ALT in µmol/L*s			
Median	0.60	0.57	0.624
IQR	0.47	0.47	
Bilirubin in µmol/L			
Median	11.00	7.00	** < 0.001**
IQR	14.00	4.00	
Ferritin in µg/L			
Median	4227.15	2079.85	**0.002**
IQR	5705.68	2768.80	

*p*-values of less than 0.05 indicated statistical significance (in bold)

*ALT* alanine aminotransferase, *aPTT* activated partial thromboplastin time, *INR* international normalized ratio, *IQR* interquartile range, *SOS* sinusoidal obstruction syndrome, *vWF* von Willebrand factor, *vWF activity* von Willebrand factor activity

**Fig. 1:**
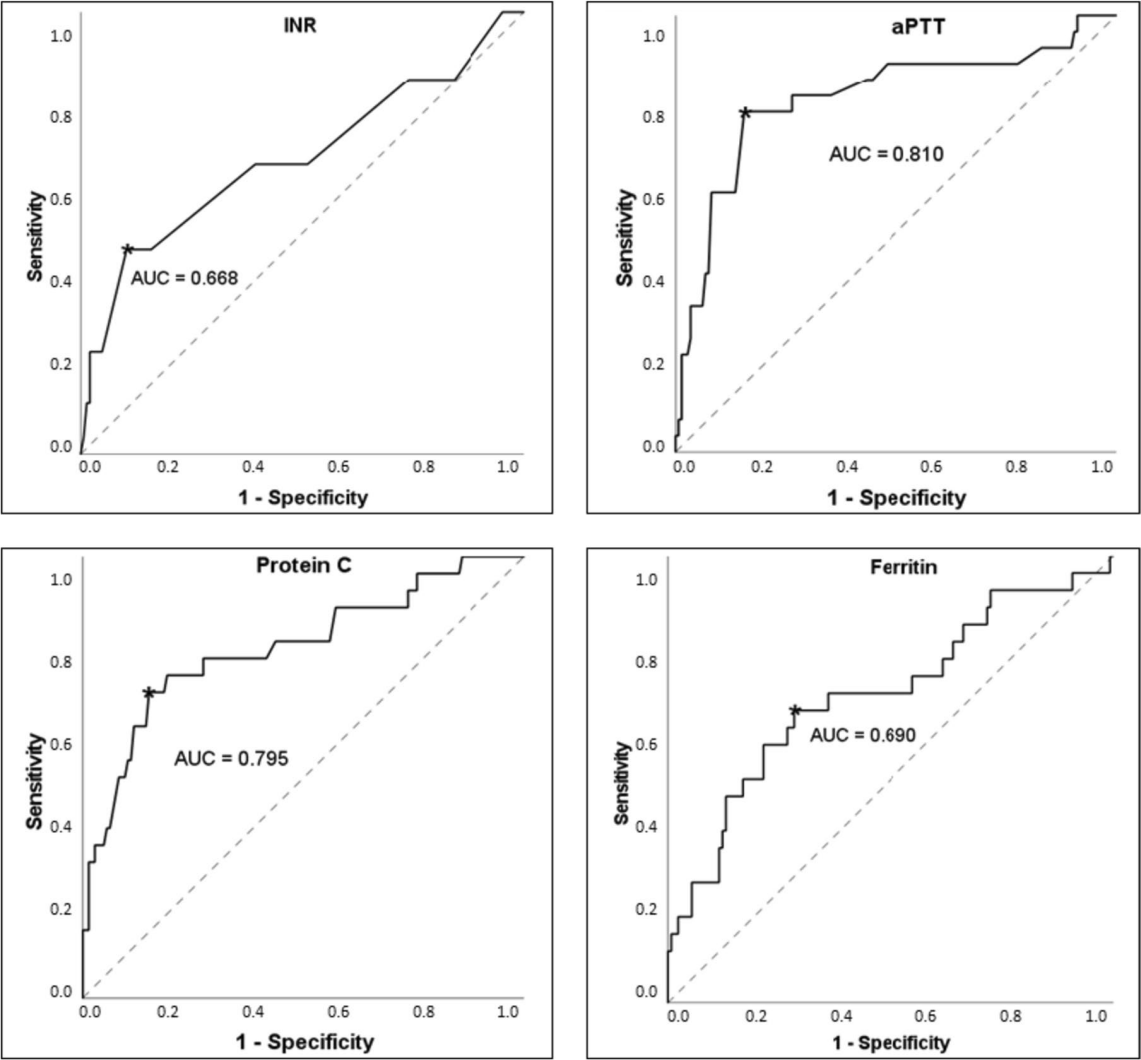
Receiver operating characteristic curves for the cut-off values of international normalized ratio (INR), activated partial thromboplastin time (aPTT), protein C, and ferritin. Best cut-off values are marked with an *; *AUC* area under the curve

We selected cut-off values, which were defined by reference values and ROC curve analyses. Figure 1 demonstrates the ROC curves of INR, aPTT, protein C, and ferritin with the cut-off values. The results of the univariate anlyses are presented in Table 3 with the corresponding *p* value, odds ratio, and 95% CI. For the values of factor XIII, vWF, VWF activity, fibrinogen, protein S, and ALT we could not find any significant results. If the coagulation parameters exceeded a certain value, such as INR ≥ 1.3, aPTT ≥ 40 s, and reptilase time ≥ 18.3 s, the SOS was diagnosed significantly more often. In addition, patients with factor VIII ≤ 80%, antithrombin III ≤ 75%, and protein C ≤ 48% showed a significantly higher incidence of SOS. We detected a significant association between the onset of SOS and the serum levels of D-dimer ≥ 315 µg/L, bilirubin ≥ 9 µmol/L, and ferritin ≥ 3100 µg/L after HSCT.

**Table 3:** Univariate analyses of laboratory parameters

Laboratory parameters	Total No.	SOS No.	Univariate analyses		
			OR	95% CI	*p*
INR					
≥ 1.3	27	12 (44.4%)	7.429	2.907–18.982	** < 0.001**
< 1.3	144	14 (9.7%)			
aPTT					
≥ 40 s	44	21 (47.7%)	19.022	6.925–52.247	** < 0.001**
< 40 s	131	6 (4.6%)			
Reptilase time					
≥ 18.3 s	64	15 (23.4%)	4.041	1.376–11.869	**0.014**
< 18.3 s	71	5 (7.0%)			
Factor VIII					
≤ 80%	18	12 (66.7%)	17.400	5.355–56.537	** < 0.001**
> 80%	97	10 (10.3%)			
Factor XIII					
≥ 75%	90	15 (16.7%)	1.511	0.621–3.677	0.386
< 75%	77	9 (11.7%)			
vWF					
≤ 170%	66	14 (21.2%)	1.713	0.720–4.079	0.272
> 170%	81	11 (13.6%)			
vWF activity					
≤ 160%	58	14 (24.1%)	2.256	0.943–5.395	0.075
> 160%	89	11 (12.4%)			
Fibrinogen					
≥ 3.5 g/L	92	16 (17.4%)	1.512	0.659–3.467	0.405
< 3.5 g/L	90	11 (12.2%)			
Antithrombin III					
≤ 75%	46	15 (32.6%)	4.960	2.109–11.664	** < 0.001**
> 75%	135	12 (8.9%)			
Protein C					
≤ 48%	40	18 (45.0%)	12.784	4.953–32.994	** < 0.001**
> 48%	133	8 (6.0%)			
Protein S					
≤ 50%	84	17 (20.2%)	2.284	0.925–5.638	0.083
> 50%	80	8 (10.0%)			
D-dimer					
≥ 315 µg/L	90	21 (23.3%)	3.601	1.371–9.461	**0.010**
< 315 µg/L	77	6 (7.8%)			
ALT					
≥ 1 µmol/L*s	30	5 (16.7%)	1.182	0.409–3.415	0.780
< 1 µmol/L*s	152	22 (14.5%)			
Bilirubin					
≥ 9 µmol/L	70	19 (27.1%)	4.843	1.986–11.810	** < 0.001**
< 9 µmol/L	112	8 (7.1%)			
Ferritin					
≥ 3100 µg/L	54	17 (31.5%)	4.799	1.965–11.721	**0.001**
< 3100 µg/L	103	9 (8.7%)			

*p*-values of less than 0.05 indicated statistical significance (in bold)

*ALT* alanine aminotransferase, *aPTT* activated partial thromboplastin time, *CI* confidence interval, *INR* international normalized ratio, *No.* number, *OR* odds ratio, *SOS* sinusoidal obstruction syndrome, *vWF* von Willebrand factor, *vWF activity* von Willebrand factor activity

We investigated nine laboratory parameters in the multivariate analysis, which were significant in the univariate analyses. After binary logistic regression and backward elimination, we identified four laboratory parameters as significant independent variables. The significant correlation between INR ≥ 1.3 after HSCT and SOS could be confirmed (OR = 8.104, 95% CI = 1.847–35.554, *p* = 0.006). In addition, aPTT ≥ 40 s before SOS was significantly associated with the onset of SOS (OR = 10.174, 95% CI = 2.722–38.020, *p* = 0.001). Protein C ≤ 48% (OR = 5.215, 95% CI = 1.390–19.562, *p* = 0.014) and ferritin ≥ 3100 µg/L (OR = 7.472, 95% CI = 1.912–29.202, *p* = 0.004) could be affirmed as significant predictive variables. Table 4 displays the significant results of the multivariate analysis.

**Table 4:** Multivariate analysis of laboratory parameters

Laboratory parameters	Multivariate analysis		
	OR	95% CI	*p*
INR ≥ 1.3	8.104	1.847–35.554	**0.006**
aPTT ≥ 40 s	10.174	2.722–38.020	**0.001**
Protein C ≤ 48%	5.215	1.390–19.562	**0.014**
Ferritin ≥ 3100 µg/L	7.472	1.912–29.202	**0.004**

* p*-values of less than 0.05 indicated statistical significance (in bold)

*aPTT* activated partial thromboplastin time, *CI* confidence interval, *INR* international normalized ratio, *OR* odds ratio

The four significant laboratory parameters can be used to predict the probability of SOS, which depends on the regression coefficients of the parameters. We calculated the probability of SOS for each combination of the existing cut-off values using logistic regression. If three of the four significant cut-off values were present, the probability of developing SOS was more than 70%. Additionally, if all four laboratory parameters were changed according to the cut-off values, the probability of SOS was 96%.

## Discussion

In this retrospective analysis, 27 out of 182 patients (14.8%) developed SOS after HSCT. Following allogeneic HSCT, the incidence was higher (19.1%) than in the autologous patient group (5.4%). Therefore, our study is in line with other publications, which report comparable incidence rates of 14–20% (Xia et al. 2021; Coppell et al. 2010). Additionally, the published lower incidence rate following autologous HSCT (8.7%) in comparison to allogeneic HSCT (12.9%) by Coppell et al. (2010), agrees with our findings. In our study, SOS was diagnosed within 1 to 28 days after HSCT (median 13 days). This is comparable to a study by Coutsouvelis et al. (2023), in which a median of 14 days after HSCT was reported. We observed a low overall mortality of 11.1% in SOS patients within 100 days following HSCT. Coutsouvelis et al. (2023) reported a similar mortality rate of 9.4%. An early treatment with defibrotide is associated with a better outcome and could be the reason for the low mortality rate in our study (Corbacioglu et al. 2004).

We found significantly higher INR values in the SOS group than in the non-SOS patient group after HSCT before diagnosis. Moreover, our study demonstrated in the univariate and the multivariate analysis (OR = 8.104, *p* = 0.006), that INR ≥ 1.3 after HSCT was significantly associated with the occurrence of SOS. A study by Roeker et al. (2019) showed that the INR values in SOS patient group were significantly higher than in non-SOS group on the day of diagnosis. In addition, a high INR ≥ 1.3 before HSCT was considered as a risk factor for the occurrence of SOS (Kloehn et al. 2022). To our knowledge, we report the association between SOS and high INR after HSCT before SOS for the first time. The INR is a blood coagulation screening test, which is particularly dependent on the coagulation factors II, V, VII, and X. Increased INR values are an indication of a bleeding tendency (Bonar et al. 2017; Favaloro and Adcock 2008). Reasons for the increased INR values before the SOS onset could be a reduced synthesis of the coagulation factors due to liver damage or a vitamin K deficiency. Activated coagulation in SOS could also lead to depletion of coagulation factors (Favaloro and Adcock 2008). In addition, an increased bleeding tendency could influence the pathogenesis of SOS, consequently hemorrhage into the Disse space is reported (Carreras and Diaz-Ricart 2011).

Coagulation parameters, such as aPTT and reptilase time were significantly prolonged in the SOS group compared to the non-SOS group. In the univariate analysis, an association between the occurrence of SOS and prolonged aPTT ≥ 40 s or reptilase time ≥ 18.3 s after HSCT could be demonstrated. Reptilase time showed no significant results in the multivariate analysis. In the multivariate analysis, we confirmed aPTT ≥ 40 s as a predictive factor for the development of SOS (OR = 10.174, *p* = 0.001). To our knowledge, there are no studies in the literature that reported a significant association between aPTT and SOS after HSCT before SOS. However, aPTT is also prolonged after the diagnosis of SOS (Jevtić et al. 2010; Sartori et al. 2005). The aPTT is a screening test to assess intrinsic blood coagulation. A prolonged aPTT is often associated with a deficiency of coagulation factors such as factor VIII, IX, XI or XII (Kershaw 2017). Therefore, the prolonged aPTT could be caused by a decrease of factor VIII, which was reduced in our study.

The median factor VIII activity was significantly lower in the SOS group than in the non-SOS group. We detected factor VIII activity ≤ 80% as a significant variable in the univariate analysis. This, however, could not be confirmed in the multivariate analysis, which agrees with Lee et al. (1998) who reported no significant differences of factor VIII activity in the groups. Nevertheless, factor VIII appears to be an important parameter in the pathogenesis of SOS. Some studies reported that factor VIII and fibrinogen are reasons for the occlusion of the sinusoidal vessels (DeLeve et al. 2002; Shulmann et al. 1987). The reduced factor VIII activity could be the result of liver damage in the context of SOS.

Antithrombin III and protein C are important anticoagulants and showed significant lower values before diagnosis in patients with SOS. In the multivariate analysis, we observed that only protein C ≤ 48% was significantly associated with the development of SOS (OR = 5.215, *p* = 0.014) and identified protein C as an independent risk factor. Iguchi et al. (2010) reported similar results in a prospective study. The deficiency of protein C can be associated with thrombotic processes, which have an influence on the pathogenesis of SOS. Protein C is synthesized in the liver and therefore it could be decreased by the liver damage (Iguchi et al. 2010; Lee et al. 1998). Thrombotic processes are also associated with increased D-dimer levels. Our study showed that patients after HSCT before SOS had significantly higher D-dimer levels than in the non-SOS patient group. Other studies were able to show similar results (Jevtic et al. 2011; Sartori et al. 2012). We observed a significant association between elevated D-dimer levels ≥ 315 µg/L and SOS in univariate analysis, but this could not be confirmed in multivariate analysis.

In the univariate analysis, bilirubin ≥ 9 µmol/L was significant associated with the occurrence of SOS. Sartori et al. (2012) also found a significantly elevated bilirubin level before diagnosis of SOS. Bilirubin is one of the pediatric EBMT criteria for diagnosing SOS. However, the presence of hyperbilirubinemia is a non-mandatory criterion (Corbacioglu et al. 2018). In a study by Corbacioglu et al. (2020), 29% of pediatric patients did not have a bilirubin level > 34 µmol/L despite SOS. This fits with the non-significant result of bilirubin ≥ 9 µmol/L in the multivariate analysis. High bilirubin levels are often a late finding in patients with SOS (Corbacioglu et al. 2018).

We could find significant increased serum levels of ferritin in the SOS patient group after HSCT before SOS. The multivariate analysis confirmed ferritin ≥ 3100 µg/L after HSCT before SOS as an independent risk factor (OR = 7.472, *p* = 0.004). High ferritin plasma concentrations after HSCT before the onset of SOS are also reported by Döring et al. (2016). The increased ferritin could be caused by iron overload, which leads to various liver diseases such as SOS (Evens et al. 2004). Additionally, ferritin is an acute-phase protein and is increased in various inflammations in the body and thus can demonstrated inflammatory processes (Armand et al. 2012). High ferritin levels before HSCT are considered as a risk factor for developing SOS (Kloehn et al. 2022; Lee et al. 2010). Therefore, increased ferritin levels are important for the occurrence of SOS before as well as after HSCT.

Factor XIII, vWF, vWF activity, fibrinogen, protein S, and ALT did not show significant *p* values in Mann–Whitney *U* test and univariate analyses. However, there are various reports on the association of these parameters with SOS in the published literature (Jevtic et al. 2011; Lee et al. 1998; Park et al. 1997; Sartori et al. 2012).

The laboratory parameters INR, aPTT, protein C, and ferritin are independent risk factors for onset of SOS and the probability of SOS can be calculated with the combination of these parameters. Patients can present different combinations of the certain cut-off values. For each combination, there is a different probability of the SOS development. If at least three of the cut-off values were present, the probability of developing SOS was more than 70%. The probability of SOS was 96%, if all four laboratory parameters are changed according to the cut-off values. To our knowledge, this is the first report in which the probability of SOS was calculated with laboratory parameters.

In conclusion, our study confirmed INR ≥ 1.3, aPTT ≥ 40 s, protein C ≤ 48%, and ferritin ≥ 3100 µg/L as predictive factors for the development of SOS and independent risk factors. To our knowledge, the significant association between the onset of SOS with increased INR (≥ 1.3) and aPTT (≥ 40 s) after HSCT is here demonstrated for the first time.

The regular measurement of INR, aPTT, protein C, and ferritin is a useful tool to predict the occurence of SOS after HSCT before SOS. These four parameters can enable earlier diagnosis and treatment, leading to an improved outcome. Other studies are necessary to validate our results, so these results can be used in clinical practice.
